# Access to ART Services: Lived Experiences and Coping Strategies of HIV-Positive Persons With Visual Impairment in Lira District, Northern Uganda

**DOI:** 10.1155/bmri/1903886

**Published:** 2025-03-19

**Authors:** Gloria Ketty Acila, Noeline Aju Ijorea, Amir Kabunga, Mercy Joy Angom, Sandra Talemwa, Patrick Ayer, Anna Grace Auma

**Affiliations:** ^1^Department of Nursing, Lira University, Lira City, Uganda; ^2^Department of Psychiatry, Lira University, Lira City, Uganda; ^3^Department of Public Health, Lira University, Lira City, Uganda

**Keywords:** coping strategies, HIV infection, lived experience, visual impairment

## Abstract

**Background:** Despite progress in the HIV/AIDS response, marginalized populations persistently face unique challenges in accessing essential healthcare services, including antiretroviral therapy. The aim of this qualitative study is to explore and understand the lived experiences of individuals living with HIV and visual impairment in Lira District, Northern Uganda, specifically focusing on their access to antiretroviral therapy services. Additionally, the study is aimed at identifying and analyzing the coping strategies employed by this population in navigating the intersectionality of HIV and visual impairment.

**Methods:** This qualitative study, conducted in Lira District, Northern Uganda, employed a descriptive phenomenological design. Thirty in-depth interviews were conducted at Lira Regional Referral Hospital, focusing on individuals living with both HIV and visual impairment. Data collection involved a semistructured interview guide, addressing key issues derived from a literature review. Thematic analysis was used for data analysis, guided by Braun and Clarke's framework.

**Results:** Participants (*N* = 30) exhibited diverse sociodemographic characteristics, with ages ranging from 19 to 68 years. A number of themes emerged during data analysis: individuals developing visual impairment before and after ART initiation. Emotional challenges, strained relationships, and perceived burdenship were common themes among participants. Limited understanding of the cause of sight loss and a heightened perceived risk of HIV infection were evident. Challenges in accessing ART services included transportation difficulties, negative attitudes from healthcare workers, and a lack of trust in community drug distribution points. Participants employed various coping strategies, including prayers/religion, reliance on social support networks, denial, acceptance, and community rehabilitation. Positive religious beliefs offered hope, while social support played a crucial role in adaptation. Community rehabilitation and support were highlighted as instrumental in aiding coping.

**Conclusion:** Despite awareness about the risk of HIV infection, significant barriers persist in accessing ART services for persons with visual impairment. Coping strategies underscore the importance of addressing psychosocial aspects. Tailored interventions, education, and policy changes are imperative to enhance inclusivity and accessibility of healthcare services for this vulnerable population in Uganda.

## 1. Introduction

HIV/AIDS remains a global health challenge, impacting millions worldwide [1]. The African region bears a substantial burden of the epidemic, with vulnerable populations facing critical barriers to healthcare access. Data from sub-Saharan Africa indicate a heightened risk of HIV infection, with men with disabilities being 1.48 times more at risk and women with disabilities 2.21 times more at risk compared to their nondisabled counterparts [1]. Despite the crucial role of antiretroviral therapy (ART) services in HIV prevention and the achievement of the 2030 treatment goals [2], limited information exists on the epidemiology of visual impairment in individuals with HIV during the ART era [3]. Visual impairment is associated with social isolation, depression, physical limitations, and premature mortality [4]. With evidence suggesting that individuals with visual impairment are particularly vulnerable to HIV, it is essential to identify effective service delivery strategies for people with disabilities, especially those with visual impairments, to enhance universal HIV prevention and treatment efforts [5]. However, despite significant progress, marginalized groups, including those with visual impairments, continue to encounter distinctive and often overlooked challenges in accessing essential healthcare services, including ART.

This study is aimed at uncovering the coping strategies employed by individuals in this demographic to overcome barriers impeding their access to ART services. When investigating the coping processes of individuals with visual impairments, it is crucial to identify elements that contribute to positive outcomes by elucidating the correlation between stressful situations and individual disabilities [6]. However, intervention studies focusing on fostering coping mechanisms and positive adaptation lack comprehensive exploration and empirical analysis of factors within the developmental processes of individuals with visual impairments who are also living with HIV [7]. Furthermore, most studies have focused on individual resilience to adversity [8], neglecting considerations for the intersection of visual impairments and HIV, highlighting the imperative need for investigations that prioritize the enhancement of social adaptation. To deepen our understanding of factors that improve the quality of life for those with visual impairments and HIV, research should meticulously identify the challenges they face and analyze the coping process. Effectively addressing these specific intervention objectives could significantly enhance the quality of life for individuals with visual impairments who are concurrently living with HIV.

Uganda, like many sub-Saharan African countries, has made commendable progress in responding to the HIV epidemic [9]. The country has implemented various programs and policies to enhance HIV testing, treatment, and care [3]. However, the experiences of individuals with visual impairments living with HIV in Uganda remain an area requiring further investigation. Northern Uganda, emerging from the shadows of conflict and displacement, faces additional complexities in providing adequate healthcare infrastructure [10]. Despite regional efforts to address HIV/AIDS, the specific needs of visually impaired individuals navigating the intersection of HIV and visual impairment may not have received sufficient attention. Additionally, limited research exists on the lived experiences of individuals managing both HIV and visual impairment, particularly in regions such as Lira District, Northern Uganda. The interplay between these two health conditions can significantly impact access to ART services, posing potential barriers to care and necessitating a deeper understanding of the challenges faced by this specific population.

## 2. Methods and Materials

### 2.1. Study Design and Setting

The research utilized a descriptive phenomenological study design, employing a qualitative approach to data collection. The study was conducted at Lira Regional Referral Hospital (LRRH) in Lira City, Northern Uganda, situated approximately 340 km north of Kampala, the capital city of Uganda. LRRH, a public regional referral hospital, is one of nine such facilities in Uganda, serving a catchment population of approximately 2,000,000 individuals. It is the only referral hospital in the subregion that offers all essential medical services, including specialized care. The hospital provides general and specialized services, including an ophthalmology clinic, and attends to approximately 1000 clients annually, with an average of 60 clients per month from Lira City and the surrounding districts. This study involved 30 in-depth interviews with HIV-positive individuals with visual impairment, focusing on their lived experiences and coping strategies in accessing ART services.

### 2.2. Sample Size Estimation

The study population comprised individuals living with HIV and experiencing visual impairment. In February 2022, 30 in-depth interviews were carried out with participants attending the ophthalmology clinic at LRRH. We consecutively selected and interviewed individuals living with HIV/AIDS and visual impairment, who had sought or were seeking care at the ophthalmology clinic of LRRH and were above 18 years old. These participants willingly consented to take part in the study.

### 2.3. Data Collection Methods and Tools

We conducted in-depth interviews lasting between 40 and 45 min to collect data. A semistructured interview guide was employed for data collection, which was developed based on a comprehensive review of the literature addressing key issues affecting individuals living with HIV and visual impairment. Throughout the interviews, research assistants frequently probed to gain a nuanced understanding of the lived experiences and coping strategies of those living with both HIV and visual.

### 2.4. Data Collection Procedures

The study was conducted with written permission obtained from the health facilities, specifically from the District Health Officer of Lira District and the Hospital Director of LRRH. In preparation for data collection, four research assistants underwent training. Data collection involved individuals living with both HIV and visual impairment, utilizing a pretested interview guide and audio recorders managed by the research assistants. The tool employed in the study is aimed at gathering information on the lived experiences of individuals dealing with visual impairment and HIV, as well as the coping strategies employed by these clients. Prior to data collection, participants were provided with a thorough explanation of the study's purpose, the methods of data collection, and the anticipated time frame. Informed consent forms were personally delivered to prospective respondents by the researchers. Upon collection, the study researcher meticulously reviewed the gathered data at the site to identify and address any missing information. Subsequently, the collected data was securely stored in a designated cupboard. The recorded data was duplicated into a computer, and any unclear recordings were carefully edited for clarity and accuracy.

### 2.5. Data Analysis

A theoretical thematic analysis framework with six stages, following Braun and Clarke's [11] recommendations, was employed using deductive analysis, with a primary focus on sensory processing difficulties. The transcriptions were carefully read and reread in sequence, with initial notes recorded in the margins. Subsequently, codes were generated in relation to the research questions and significant aspects of the data. These codes were then examined to identify underlying themes. Data collection occurred incrementally through successive interviews, leading to the emergence of themes. Additional interview data either enriched existing themes or contributed to the development of new ones until saturation was achieved. Participants were given the opportunity to provide feedback on the summary of themes during their interviews and share additional thoughts via email. No participants proposed any alterations or amendments, and those who provided comments confirmed that the themes accurately reflected their experiences. Following this, a thematic map was constructed to address the research questions, and the themes underwent a comprehensive review and refinement to form an overarching analysis.

Lincoln and Guba's framework was employed to evaluate the rigor and trustworthiness of the qualitative data, focusing on credibility, dependability, confirmability, and transferability [12]. To establish data credibility, extended engagement with participants was conducted, with interviews lasting approximately 45 min. The researchers engaged in self-reflection before the study, bracketing their own thoughts and emotions to minimize researcher reflexivity.

Data dependability was ensured by maintaining comprehensive, detailed notes, including verbatim participant statements (documentation), and by conducting member checking, wherein participants received feedback on the interpretations of their interviews. Additionally, peer review was conducted at each phase of the study including coding and theme development in collaboration with the researchers. Codes and themes were finalized upon reaching an intercoder agreement.

## 3. Results

### 3.1. Sociodemographic Characteristics of the Respondents

We conducted interviews with a total of 30 individuals who are living with both HIV and visual impairment. Out of the 30 participants interviewed, 13 were male and 17 were female, with ages ranging from 19 to 68 years old. The participants' occupations were diverse, including 18 peasant farmers, 3 students, and 9 casual laborers (refer to Table 1 for details).

### 3.2. Participant's Experience of Being Both Visually Impaired and Living With HIV

During the process of data analysis, two distinct categories emerged: individuals who developed visual impairment subsequent to the initiation of ART and those who acquired visual impairment after commencing ART. A majority of the study participants (16 out of 30) experienced visual impairment prior to embarking on ART care or discovering their HIV status. Conversely, 14 participants developed visual impairment after becoming aware of their HIV-positive status and commencing ART.

Nevertheless, both categories appeared to share similar experiences. Participants in both groups expressed encountering challenges associated with being HIV positive and coping with the dual conditions. They reported feelings of disappointment, disorganization, disorientation, and expressing discontent with life due to the added burden of visual impairment following the onset of HIV infection. They perceived the situation as unjust and unsettling, grappling with the difficulties of visual impairment. Moreover, these participants expressed a sense of being burdensome to others, relying on external assistance for a significant portion of their needs.

… I have been such a healthy person, not depending on anyone, life was good because I could take care of all my needs, until 2002 when I realized that I was unable to see properly around November, I started feeling eye pain and serious headache, that was when I was taken to the doctor who told me I was developing an eye problem, of course, I felt bad that I almost blamed my wife for my woes. (60–63-year-old male)

Another participant put it this way

… losing my sight has been one of my nightmares, being unable to see, depending on others. Things worsened when they tested me and found me HIV positive, it felt like a double tragedy, I just wanted to die, life was useless. (40–43-year-old female)

The participants expressed the sentiment that the ARVs had already imposed a considerable burden, and consequently, experiencing visual impairment added an additional layer of hardship. This observation is evident in the following excerpt.

Another participant said

… As if it was not already bad enough to be HIV positive, taking drugs every day and having to live with the side effects of the drugs, and then this fateful day, June 2017 when I was waiting to board by the roadside when a speeding vehicle sprayed some stones into my eyes which left injuries in my eyes, I felt a lot of pain, went to the hospital and the Doctor told me that I would need to be operated to remove the stones and have my vision rescued, I thought I would be ok, but I have never felt better, life has become very disappointing and not livable. (30–35-year-old male)

A 29-year-old female added

… As if being HIV positive is not enough, I wonder what God wants from me by making me blind on top of this. (20–29-year-old female)

The emotional challenges had a negative impact on relationships with family members. Respondents perceived that their visual impairment strained these relationships, leading to heightened personal dependence and a shift in relationship dynamics. One participant articulated this sentiment as follows:

The loss of vision cost me my relationship with family and peers. It seems I was unpleasant, I can tell it is a difficult journey…. (20–23-year-old male)

Nevertheless, certain participants reported being born with the virus and had been on ART throughout their lives. However, they experienced visual impairment later, after initiating ART. They conveyed a lack of awareness regarding being free of the virus, but did express challenges associated with visual impairment. One respondent elucidated:

… I was told by my parents that I was given birth to the virus. It got transmitted to me during birth which occurred on the way due to the far distance of the hospital from home. (20–25-year-old female)

The majority of participants conveyed sentiments of despair, expressing concerns about their well-being and grappling with the challenges of being both visually impaired and HIV positive. They articulated feelings of unworthiness and an inability to assist others, relying on others for support entirely.

… I felt very bad. I felt I was the unluckiest person because why would God make my eyes stop seeing well after my parents died and left me with no one to pay for my school f? es. I had to drop out of school that I ended up doing what I'm doing now. I have realized I now need someone to depend on, yet I am the sole breadwinner for my wife and children, I am scared of how my family will continue to survive without my support. (20–25-year-old male)

Another participant stated it this way:

… I did not feel good because, after all, that I have been through, including the operation, I thought that I would regain my sight like I used to but to my surprise, here I am with poor vision and helplessness. I am scared now because anything can happen to me like being hit by a car and dying, I am not sure how I will survive this but I want to keep the hope alive. I am now scared of how I will continue to have my medication in time without any help, I am scared of defaulting drugs and becoming weaker. (30–43-year-old female)

As a result of being visually impaired and living with HIV, some respondents harbored the belief that life was effectively over, expressing a reluctance to continue living. Their self-esteem and confidence were severely undermined, and they perceived their circumstances as inescapable.

Loss of vision stopped me from going out; I was very sad and depressed. I felt sorry for myself and believed that was the end of it because I had HIV and not because I was blind. Fear after fear and I knew I was dead. (20–27-year-old female)

Another said:

When I became blind yet I had this disease (HIV), was broken just like a broken toy…. What happens to a broken toy, it is thrown in the dust and nobody wants to know you.

### 3.3. Perceived Cause of Sight Loss

Participants exhibited limited understanding of the cause of their vision loss. Some believed it was attributable to the side effects of medication, while others considered the possibility of a supernatural influence. One participant articulated this perspective as follows:

… I think that my long stay on ART contributed to my poor vision since the doctor also said that different people react to the ARVs differently, some of them react by being blind, depending on how you adapt to it. (30–32-year-old female)

Other participants held the belief that supernatural forces were responsible for their vision loss, as they were unable to provide a rational explanation for the phenomenon. This viewpoint was illustrated by one participant in the following manner:

… But somehow later, I even felt deep inside me that maybe I was just bewitched by someone. I pray every day that God should defeat the devil causing this problem so I can become better. (40–46-year-old male)

### 3.4. Perceived Risk of HIV Infection

Participants demonstrated a strong understanding of the various modes of HIV transmission. They conveyed a perception that disabled individuals face an elevated risk of contracting HIV. Instances cited included sexual assault, challenges in social life such as being intoxicated and unable to control their sexual behavior due to dependence on others, and sexual abuse by those upon whom they rely. These points are exemplified in the following extracts:

… People with disability have a higher chance of getting HIV because they are at a disadvantage of being weak, being visually impaired is a very compromising condition. Anyone can take advantage of you at any time, even the person you trust. (30–40-year-old female)

Another participant expressed it this way:

… People with disability also have feelings and want to have sex too, they also want to drink irresponsibly and this puts them at risk of getting infected by abled people who can easily overpower them due to their condition and rape them. it may be worse when both are drunk. (20–30-year old female)

Another one added:

… People with disability who drink alcohol and are irresponsible can easily be raped and get infected with HIV, they are, therefore, at a greater risk of HIV infection compared to those with intact sight. (20–30-year-old female)

### 3.5. Challenges Faced by the Visually Impaired

Participants encountered various challenges associated with their conditions of HIV and visual impairment. These challenges include the following.

#### 3.5.1. Access to ART Services by Participants

The study participants encountered difficulties in accessing transportation to and from health centers, often requiring an extended period of walking to reach the healthcare facility. On average, they spent approximately 2 h on this journey, with some opting to use bicycles or alternative means of transport. Participants highlighted that while they generally have access to medication once they reach the facility, the primary obstacle lies in the process of reaching it.

… I always use a boda-boda to move to the health facility and sometimes when I have no money, I even walk on foot to the hospital to get my ART services. When I walk, I spend about 2 hours moving to and from the health facility. (40–50-year-old male)

Community drug distribution points (CDDPs) and village health teams (VHTs) have not been identified as integral components of ART access points in most villages. However, there is a consensus among community members that incorporating these entities into the community would be highly beneficial. This inclusion is perceived as a means to alleviate the transportation challenges faced by individuals with disabilities, including the visually impaired.

#### 3.5.2. Negative Attitude by the Health Workers

While a majority of the participants conveyed positive sentiments regarding healthcare professionals at the hospital, it is important to note that some participants experienced harsh treatment that acts as a deterrent, discouraging them from accessing the facility for ART care. This impediment has resulted in irregular utilization of ART services, thereby hindering their ability to adhere to the prescribed medications as expected.

… I would say that they are good but when their days are not good, they tend to be harsh in that when you look young, they can give you hard time by telling you how you have failed to listen and that is why you are in such a condition, they make you feel like it is your fault to be HIV positive and blind at the same time. (0–26-year-old female)

Another participant expressed his concern this way:

… I have been on and off the treatment because of how those nurses at the ART clinic treat me and also my problems. The staff in most cases are not fair because they start by serving the people who come late and leave the ones who came so early, that is annoying and discourages me from going to the ART clinic because we all leave our homes to come for a refill and therefore no one should be treated or taken as exceptional unless one is sick. (20–30-year-old male)

Conversely, some participants reported positive experiences with healthcare providers, highlighting the warm reception they received. They expressed gratitude towards those delivering ART services to the visually impaired, emphasizing that the quality of care was commendable. This positive experience fostered a sense of comfort, prompting them to willingly return to the facility, even if it meant walking, as they were confident in receiving the necessary services.

… The nurse who declared my HIV status was very nice and reassuring in the way she handles things. She did not waste time because she did everything very fast enough. She was not segregated because she treated everyone as differently as they are and also understood my situation fully.

#### 3.5.3. Lack of Trust and Knowledge About CDDPs and the VHTs

Certain clients expressed specific preferences regarding who is authorized to collect their medication, limiting access only to those individuals aware of their HIV status. Many have refrained from disclosing their status to a broader audience. Additionally, a notable portion of these individuals lacks knowledge about the identities of CDDPs and VHTs. Some exhibit mistrust towards VHTs, hesitating to share their information with them.

… There was a time a nurse gave us a talk on how we can access our drugs through the CDDPs but I have not yet discovered how to get them and enroll. The nurses also said we can access the drug from the VHTs but those people are not trustworthy and may spill out the information that I am HIV positive to other people in the community; do not want everyone to know my status. (20–29-year-old female)

Another participant put it this way:

… There is no other way that I always obtain my ARVs apart from physically going to the health facility. I would say I don't have any experience with the health care VHTs, it is only my wife who knows my status and it is our secret because even my children have not disclosed it to them. It is therefore only my wife who can pick up my drugs other than myself. (40–45-year-old male)

Another participant expressed how they do not know CDDPs and VHTs:

… I am still new to ART care so I have not heard about or known about VHTs and CDDPs bringing drugs closer to us. I think there is a need to let me know more information about this that I have not heard about. (20–28-year-old female)

Despite the fact that some participants found themselves without alternatives, lacking anyone to collect their medication on their behalf, they made concerted efforts to reach the facility. However, in instances where they were unable to do so, it resulted in missed doses, adversely impacting their adherence to the prescribed treatment.

… There are no other ways that I obtain or get my ARVs apart from physically going to the facility. This has affected my continuity of taking drugs, sometimes I miss treatment until can go and pick up the drugs. (30–33-year-old male)

A small number of participants mentioned alternative avenues for accessing ART, such as VHTs and CDDPs, which are in closer proximity to them. These VHTs and CDDPs play a crucial role in bringing ART services directly to the community, thereby alleviating transportation and other challenges associated with accessing the medication.

… I have interacted with a VHT who also confirmed to me that we shall have a community drug distribution point, this will ease our access to ART, and I cannot wait for this to come. Already I have benefitted from the VHT services, the CDDP may even be better. (50–52-year-old male)

### 3.6. Coping Strategies by Participants

This study investigated the coping mechanisms employed by individuals who are HIV positive and visually impaired in the Lira District. The participants managed to adapt and cope with the dual challenges of HIV and visual impairment by employing various strategies including the following.

#### 3.6.1. Prayers/Religion/Spirituality

Participants articulated coping strategies and adaptations related to visual impairment and HIV status, particularly in the context of their spiritual and religious beliefs. In general, maintaining positive religious beliefs offered a sense of hope for continued living. The majority of participants sought solace in prayers, religious practices, and engaging in religious activities as mechanisms to navigate the dual challenges of HIV and disability. Many reported that connecting through prayer with supreme powers aided in their adjustment and facilitated the pursuit of a normal life. One respondent illustrated this sentiment as follows:

… I have decided to commit myself to God, I implore others to take this opportunity to make God their only consoler I have always been very close to the church since I wedded. I left worrying about death. (30–32-year-old female)

Another participant said:

My prayer every day is simple; Lord thanks for everything, I hope that you will help me in this journey, and with your help, I will these two wars (visual impairment and HIV).

#### 3.6.2. Social Support System

Another coping strategy embraced by visually impaired individuals living with HIV was social support. Support from families, friends, and community members played a crucial role in assisting them to adapt and continue with their lives. One participant expressed this sentiment in the following manner:

… I have decided to work hard to keep my mind away from worries, my Good parents have always stood by my side in terms of guidance and counseling. They normally pay visits to my home every Saturday, When I am stressed, I talk to my wife, and she makes me feel wanted like I have a reason to live again, and I end up feeling better thereafter. (40–46-year-old male)

Another one said:

… My wife reminds me also reminds me about my refill appointment date and also the time for my medication so that I do not forget to take my drugs. (30–36-year-old male)

One participant praises the mother for the support and the school counselor…

… My mother carries the heaviest load for me, she supports me emotionally and psychologically, and she even replaces my glasses. When my eye condition worsens, my colleagues or friends copy my notes. Our guidance and counseling teacher always helped me in case I am emotionally or psychologically oppressed. (19-year-old female)

… My uncle sometimes calls to ask me about my next appointment date to ensure that I do not forget about it. (20–22-year-old female)

#### 3.6.3. Denial

Certain participants were profoundly impacted and struggled to come to terms with the dual challenge of being both HIV positive and visually impaired. This difficulty was exacerbated by the negative societal and institutional attitudes towards individuals with disabilities. The fear of social scrutiny and a sense of helplessness stemmed from the prevailing negative perceptions surrounding visual impairment and HIV. One respondent vividly illustrated this sentiment:

… I had known for some time that there was something and I could not accept it. I could not accept that I had HIV and now I was blind, I had to walk everywhere, got angry with people because it was everyone else's fault…. (30–37-year-old female)

Another respondent had the following to say:

First of all I could not believe HIV positive, even when I became blind, I could not believe it. Trust I believe I will regain my sight. Don't you believe in miracles? She asked. What you believe is what you get. (20–26-year-old female)

#### 3.6.4. Acceptance

Denial and acceptance were contrasting coping mechanisms, with some participants struggling to come to terms with their dual conditions. The participant conveyed that acceptance of reality led to an increased sense of confidence. This, in turn, had a positive impact on mental well-being, facilitating the process of acceptance, as illustrated by one respondent:

Life has to go and you have to accept these things (HIV and visual impairment). Once I accepted I got used and I just moved on. Their other people like me if not worse than me. I know I can still do many daily tasks I put my mind to. (40–42-year-old female)

Considering HIV and visual impairment as challenges that affect many people and accepting this status was identified as a crucial coping strategy, as expressed by another participant:

At first I could not believe that I was both HIV positive and blind at the same time, but later I told myself I can manage and go through this, luckily am not the first person nor the last, let me accept. (40–48-year-old male)

#### 3.6.5. Community Rehabilitation

Community rehabilitation and support have proven instrumental in aiding individuals with disabilities living with HIV to cope with the challenges presented by both visual impairment and the virus. This is exemplified in the following excerpts:

The people in the community have been good to me. Some people have been key people to create awareness of HIV-positive people and are the most important and they have a great role to play to the communities in alleviating the situation. I can trust and rely on them for support. (40–50-year-old male)

Another one said:

We have been trained most time on how to live independently at the rehabilitation center. They also teach us the dangers and prevention of HIV/AIDs. Some days the officials at the rehabilitation bring to handle health-related issues including HIV and how to live with it. (30–33-year-old female)

## 4. Discussion

We aimed to explore and understand the lived experiences of individuals living with HIV and visual impairment in Lira District, Northern Uganda, specifically focusing on their access to ART services. Additionally, the study is aimed at identifying and analyzing the coping strategies employed by this population in navigating the intersectionality of HIV and visual impairment in the Ophthalmology Clinic at LRRH in Lira City. Upon reflecting on the findings, it became evident that these individuals experienced a spectrum of emotions and behaviors. They reported feelings such as disappointment, disorganization, disorientation, and even a sense of despair. These psychological impacts are likely a consequence of the stigma associated with disability and HIV itself, a phenomenon observed in many people with disabilities [13]. Research examining the combined impact of vision loss and HIV on psychological well-being indicates impairments in self-esteem, challenges in interpersonal relationships, and reduced participation in social activities [14]. Our findings closely align with the outcomes of prior studies [15]. The presence of comorbid conditions is likely to impede the overall quality of life for individuals with disabilities [14]. Consequently, there is a compelling need to integrate mental health rehabilitation into the spectrum of HIV/ART care services in Uganda. This holistic approach acknowledges the psychological challenges faced by individuals with dual conditions and strives to enhance their overall well-being.

Our study indicates that participants exhibited a high level of awareness regarding the perceived risk of HIV infection. Contrary to studies in sub-Saharan Africa, which suggest an increased relative risk for HIV infection among persons with disabilities [16], our findings reveal that individuals with disabilities possess substantial knowledge about the perceived risk of HIV. Consistent with our results, research also demonstrated that people with disabilities were well informed about the risk factors associated with HIV infection [17]. This knowledge could be attributed to the ongoing awareness programs and campaigns implemented by the Ugandan government. However, our results diverge from earlier studies that reported lower levels of knowledge about the risks of HIV infection among people with disabilities [18]. The disparity between our findings and those of previous studies may be attributed to variations in methodologies and sample sizes.

Our findings indicate that individuals with disabilities who are also living with HIV encounter various challenges, including negative attitudes from healthcare professionals, transportation difficulties, and a lack of trust in and knowledge about CDDPs and VHTs. The physical accessibility of ART services emerges as a significant factor contributing to the limited availability of services. Consequently, there is a heightened risk of nonadherence to treatment and a diminished quality of life for clients infected with HIV, particularly those with disabilities [17]. Our results highlight a knowledge gap concerning the causes of sight loss, which aligns with previous research on obstacles to accessing general healthcare services [19]. The current study specifically identifies a deficiency in knowledge and inadequate treatment of individuals with disabilities by healthcare professionals, along with the presence of healthcare facilities and services that are unfriendly to those with disabilities [19]. Comparable results were found in studies conducted in India [4] and South Africa [20].

Our findings reveal that participants utilized a combination of maladaptive and adaptive coping strategies. A significant number of participants expressed that prayers, religion, and spirituality played a crucial role in their lives, particularly in addressing the dual challenges of disability and HIV. The use of prayers or religion/spirituality emerged as a significant factor in the lives of people living with HIV, consistent with previous research indicating the importance of prayers as a survival mechanism for mental health outcomes [21]. Corroborating our results, existing studies have highlighted the significance of prayers in promoting mental well-being [21]. Additionally, research aligns with our findings, emphasizing that prayers are commonly employed for health-related reasons [22]. As such, it is imperative to incorporate prayers or religion/spirituality as an integral component of patient management.

Our findings highlight the significance of social support as a crucial coping strategy employed by individuals who are visually impaired and living with HIV. Support from family, friends, and peers emerges as a pivotal mediating factor in effectively managing stressful events [21]. Social support plays a key role in mitigating the stress associated with both the disease and the disability itself. It fosters optimism and contributes to better adherence to medication [21]. In alignment with the outcomes of our study, family support stands out as a valuable resource in facilitating access to and adherence to medication [21]. This is consistent with existing research, indicating that patients with support from their families are more likely to access and adhere to medication compared to those without such support [23].

Our findings suggest that community rehabilitation has proven effective in addressing the challenges associated with visual impairment and HIV. A majority of the participants have embraced community engagement through participation in social groups, recognizing the importance of family and relational support. These study results align with previous research conducted in South Africa, which advocated for community rehabilitation as a means to assist individuals with disabilities in navigating the concurrent challenges of disability and HIV [24]. Managing dual challenges may appear intricate, demanding cognitive and behavioral skills alongside social support. There is compelling evidence indicating that support groups play a pivotal role in enhancing the quality of life for individuals living with HIV [25]. Consequently, individuals facing both HIV infection and visual impairment stand to benefit from community-based rehabilitation, social support, and home-based care.

## 5. Conclusion

While our findings reveal a commendable level of awareness about the risk of HIV infection among individuals with disabilities, significant barriers to accessing ART services persist, including negative attitudes from healthcare professionals and physical accessibility issues. The coping strategies employed by participants, ranging from prayers and spirituality to strong social support networks and community rehabilitation, emphasize the importance of addressing the psychosocial aspects of living with dual conditions. Recognizing the effectiveness of community-based interventions, particularly support groups, suggests avenues for enhancing the overall quality of life for individuals facing the intricate challenges of HIV and visual impairment. This study underscores the imperative for tailored interventions, education, and policy changes to improve the inclusivity and accessibility of healthcare services for this vulnerable population in Uganda.

### 5.1. Clinical Implication

The study highlights the urgent need for healthcare systems to adopt inclusive, patient-centered approaches to ART service delivery for individuals with visual impairment. Addressing transportation barriers, integrating CDDPs, and training healthcare providers on disability-sensitive care can improve access and adherence. Psychological support and counseling should also be prioritized to mitigate the emotional distress associated with dual conditions. Strengthening social support systems and ensuring equitable, respectful treatment in healthcare facilities will enhance the overall well-being and quality of life for this vulnerable population.

### 5.2. Limitations

The study has certain limitations. Participants were recruited through purposive sampling, which has its own constraints. The individuals involved in this study were exclusively sourced from Lira City. Consequently, the narrative presented in this sample may not accurately represent individuals who did not participate in the study. Additionally, participants were asked sensitive questions pertaining to visual impairment and HIV, introducing the possibility of socially acceptable responses. However, this concern was mitigated by providing participants with clear expectations, establishing rapport, and encouraging them to ask questions.

## Figures and Tables

**Table 1:** Sociodemographic characteristics of the participants (*N* = 30).

**Variables**	**Participants ** **n** = 30** (%)**
*Age in years*	
0–20	3 (10)
21–40	11 (37)
41–60	5 (17)
60 and above	11 (36)
*Gender*	
Male	13 (43)
Female	17 (57)
*Occupation*	
PF	18 (60)
Student	3 (10)
Casual laborer	9 (30)
*Duration on ART*	
0–1 year	5 (17)
1–10 years	13 (43)
11–20 years	4 (13)
20 years and above	8 (27)

## Data Availability

The datasets used and/or analyzed during the study are available from the corresponding author on reasonable request.
